# The Basal Forebrain Cholinergic Nuclei and Their Relevance to Schizophrenia and Other Psychotic Disorders

**DOI:** 10.3389/fpsyt.2022.909961

**Published:** 2022-07-06

**Authors:** Sofia Eickhoff, Leon Franzen, Alexandra Korda, Helena Rogg, Valerie-Noelle Trulley, Stefan Borgwardt, Mihai Avram

**Affiliations:** Translational Psychiatry, Department of Psychiatry and Psychotherapy, University of Lübeck, Lübeck, Germany

**Keywords:** basal forebrain cholinergic nuclei (BFCN), psychotic disorders (incl schizophrenia), neuroimaging, acetylcholine (ACh), muscarinic acetylcholine receptor (mAChR), nicotine acetylcholine receptor (nAChR)

## Abstract

The basal forebrain cholinergic nuclei (BFCN) provide the main cholinergic input to prefrontal cortices, the hippocampi, and amygdala. These structures are highly relevant for the regulation and maintenance of many cognitive functions, such as attention and memory. *In vivo* neuroimaging studies reported alterations of the cholinergic system in psychotic disorders. Particularly, a downregulation of nicotinic and muscarinic acetylcholine receptors has been found. Crucially, such alterations in neurotransmission have been associated with cognitive impairments and positive and negative symptoms. Recent pharmacological studies support these findings, as they demonstrated an association between the manipulation of cholinergic transmission and an attenuation in symptom severity. Targeting acetylcholine receptors has therefore become a focus for the development of novel psychopharmacological drugs. However, many open questions remain. For instance, it remains elusive what causes such alterations in neurotransmission. While evidence supports the idea that BFCN structural integrity is altered in schizophrenia, it remains to be determined whether this is also present in other psychotic disorders. Furthermore, it is unclear when throughout the course of the disorder these alterations make their appearance and whether they reflect changes in the BFCN alone or rather aberrant interactions between the BFCN and other brain areas. In this review, the specific role of the BFCN and their projections are discussed from a neuroimaging perspective and with a focus on psychotic disorders alongside future directions. These directions set the stage for the development of new treatment targets for psychotic disorders.

## Introduction

Psychosis reflects a clinical syndrome encompassing several symptoms such as perceptual alterations (e.g., hallucinations), abnormal thinking (e.g., delusions), and bizarre behaviors. It is a defining characteristic of several psychotic disorders, such as schizophrenia, but may also be present in other psychiatric (e.g., major depression) and neurological disorders (e.g., Alzheimer's disease) (1). The treatment of psychotic disorders, especially schizophrenia, remains challenging to date, which is predominantly due to the limited efficacy of antipsychotic drugs to attenuate negative symptoms and cognitive impairments which often co-occur with psychotic symptoms (e.g., hallucinations and delusions) (2). Particularly, cognitive impairments (e.g., decision-making, memory, and attention) are highly relevant, as they are predictive of the onset and severity of psychotic symptoms as well as the functional outcome of the disorder (3). In fact, current evidence indicates that cognitive impairment is relevant both in the early and later stages of psychotic disorders. First, evidence demonstrates that impaired cognition is already present before the psychosis onset (4), which suggests that cognitive impairment is not simply a side effect of psychosis, as it precedes it. Indeed, working models of schizophrenia highlight that typical signs and symptoms of schizophrenia may be secondary to alterations in more fundamental cognitive functioning (5). Second, evidence indicates that at least some cognitive functions worsen over time (6), indicating the existence of effects of chronic-progressive processes in psychotic disorders. However, it is unknown which chronic-progressive processes lead to such worsening in cognition, with evidence supporting the influence of both life-style factors and long-term effects of medication (7).

Current models of psychosis are dominated by dopaminergic and glutamatergic hypotheses, mainly indicating dysfunctions in dopaminergic and glutamatergic neurotransmission. While glutamatergic dysfunction is proposed to largely relate to genetic influences, dopaminergic dysfunction is more likely elicited downstream of abnormalities in other systems such as the glutamatergic system (8). Regarding dopaminergic dysfunction, on the one hand, neuroimaging and pharmacological studies consistently report alterations in dopamine synthesis capacity and storage and dopamine release in the dorsal striatum. On the other hand, a reduction in psychotic symptom severity after the administration of dopamine (D2) antagonists has been reported as well (9).

Beyond changes in neurotransmission, neuroimaging studies have consistently demonstrated alterations in thalamocortical interactions in schizophrenia and other psychotic disorders. Specifically, several studies reported hyperconnectivity between the thalamus and sensorimotor cortices as well as hypoconnectivity between the thalamus and prefrontal-limbic regions in patients (10, 11). These aberrant patterns of connectivity have been related to specific symptom dimensions in patients with schizophrenia, including psychotic symptoms and cognitive impairment (12, 13). Crucially, thalamocortical dysconnectivity has been linked to aberrant dopaminergic transmission in chronic patients with schizophrenia, indicating a pathophysiological link; perhaps reflecting modulatory effects of aberrant striatal dopamine on cortico-striato-thalamo-cortical (CSTC) circuitry (14).

However, several other modulatory sources may influence CSTC circuitry and generate or maintain symptoms, including the cholinergic system. Indeed, dopaminergic and cholinergic neurons have reciprocal relationships (15). For instance, corticostriatal neurons synapse upon cholinergic interneurons, which in turn modulate dopamine neurons, thereby controlling striatal dopamine release (9). Intriguingly, mounting evidence indicates that the cholinergic system may play an important role in schizophrenia and other psychotic disorders (16, 17). This system includes the basal forebrain cholinergic nuclei (BFCN) and their extensive projections to the cortex (18). It is involved in regulating several important cognitive functions, which are often deficient in psychotic disorders (19, 20). Current evidence of both post-mortem and *in vivo* imaging studies—either using single photon emission computer tomography (SPECT), positron emission tomography (PET) or magnetic resonance imaging (MRI)—have demonstrated alterations of the basal forebrain cholinergic system in psychotic disorders, including changes in cholinergic receptor availability and structural changes of the BFCN (15–18). Furthermore, pharmacological modulation of this system with xanomeline, a muscarinic acetylcholine receptor agonist, appears to lead to improved cognitive functions and attenuated positive and negative symptoms in patients with schizophrenia (16, 21).

However, it remains elusive if the BFCN alterations in psychotic disorders are a result of pathophysiological processes that appear early on in the course of the disorder or result from long-term treatment with antipsychotics paired with chronicity. Furthermore, it is currently unknown whether these alterations are schizophrenia or psychosis specific, as most studies were conducted in patients with schizophrenia (22–24).

In this narrative review, first, we briefly summarize the anatomic organization of the basal forebrain cholinergic system as well as its receptors, neurotransmission, and its role in cognition in healthy controls. This is crucial for increasing the comprehensibility of the subsequently reviewed findings. These findings comprise available neuroimaging findings regarding alterations of the cholinergic system in psychosis. To encourage the development of new treatment options for psychotic disorders, we finally discuss their potential clinical implications along with directions for future research.

## Neuroanatomical Organization of the BFCN

The BFCN are the main source of acetylcholine in the central nervous system and are organized into four distinct cell groups: Ch1 = the medial septal nucleus, Ch2 = the vertical limb of the diagonal band of Broca, Ch3 = the horizontal limb of the diagonal band of broca, and Ch4 = the basal magnocellular complex that includes the substantia innominiata, the nucleus basalis of Meynert, the magnocellular preoptic nucleus, and the ventral pallidum (18). These cell groups can modulate the activity of neurons located in the prefrontal cortices, hippocampi, and amygdala primarily by activating cholinergic receptors (16). These projections are organized in clusters, based on both topographical and functional principles, which allow for a spatially selective modulation of individual or joint cortical areas (25–27). Briefly, more medial-anterior located neurons of the BFCN project to the medial frontal cortex, while the substantia innominata, the nucleus basalis of Meynert, and the diagonal band neurons project to dorsal regions of prefrontal cortical areas. More lateral parts of the BFCN project to more ventral regions of the prefrontal cortex and rostral parts of the BFCN project to both superficial and deep layers of the frontal cortex, in contrast to caudal areas of the BFCN which project to deep layers of the cortex (28). Lateral- and posterior- parts of the BFCN project to lateral cortical and subcortical areas. One main exception to this general topographical organization is that cholinergic cell groups in the medial septal nucleus and the vertical limb of the diagonal band of broca project posteriorly to the hippocampus and the entorhinal cortex (29). Regarding the functional relevance of these projections, it has been proposed that cortical connections to the prefrontal cortex (PFC) mediate decision-making, planning, and ascribing salience, while projections to the hippocampus and amygdala influence attention, memory, fear, and stress (30).

## Cholinergic Receptors and Transmission

Acetylcholine (ACh), the main neurotransmitter of the basal forebrain cholinergic system, acts on two families of receptors: nicotinic acetylcholine receptors (nAChRs) and muscarinic acetylcholine receptors (mAChRs). NAChRs are ligand-gated ion channels, while mAChRs are G-protein-coupled receptors. Ligand-gated ion channels are integral membrane proteins, which allow the regulated flow of selected ions across the plasma membrane (31), whereas G protein–coupled receptors bind ligands outside the cell to trigger events inside the cell by selectively binding and activating specific G proteins (32). These receptor families can be further divided into several subtypes, for instance nAChRs in the brain can be differentiated into 12 subtypes (α2–10 and β2–4). Each subtype is able to act by itself or in combination with others. The most common nAChR subtypes in the brain are α4β2 and α7. Similarly, the second family of receptors, mAChRs, consists of five subtypes (M1–M5). M1, M3, and M5 are excitatory, G-protein-coupled receptors, whereas M2 and M4 are inhibitory protein-coupled receptors (33).

Evidence suggests that ACh transmission includes both tonic and phasic release, however, the former has recently been questioned (34). While tonic release reflects volume transmission in minutes and is related to global brain-states including arousal, phasic release only takes milliseconds up to seconds and is thought to directly modulate cognitive and behavioral processes, such as attention (18, 35). It is the timing of ACh release that is particularly important for attention-related cognitive operations. For instance, animal studies have shown that selective cholinergic activation in the PFC on the scale of milliseconds to seconds is linked to cue detection and cue-triggered changes in goal-driven attention (27). In support, new evidence from real-time amperometric recordings in rats suggests that cholinergic signaling in attentional contexts is rapid, phasic, transient, probably synaptic, and can be trial and event specific (34).

Importantly, the cholinergic system also interacts with other systems, particularly with the dopaminergic system (15). In this context, cholinergic projections may have a modulatory, rather than an excitatory or inhibitory effect. For instance, an interplay between neurotransmitter systems is relevant for pathophysiological processes seen in Parkinson's disease (18). Specifically, while the main motor deficits associated with Parkinson's disease arise from the loss of dopaminergic cells in the substantia nigra pars compacta, a parallel loss of cholinergic cells leads to an increase in cognitive decline. A similar interaction, albeit in the opposite direction, has been suggested for Alzheimer's disease (36). Accordingly, since the dopaminergic system is altered in psychotic disorders (37), it is conceivable that interactions between the dopaminergic and cholinergic systems are also relevant for the pathophysiology of psychotic disorders. Indeed, Scarr et al. (17) suggested that changes in nAChRs and M1-mAChRs may be involved in dopaminergic dysregulation in patients with schizophrenia.

## Role of the Cholinergic System in Cognition

The relevance of the cholinergic system for cognitive functioning (e.g., attention, memory, decision-making, and overall task-performance) has been demonstrated both by preclinical human and animal studies.

Preclinical human and animal studies provide evidence from two lines of research. The first line comes from lesioning studies. For instance, lesioning the BFCN projections to the cortex via deafferentation leads to selective impairment of attentional functions in animals (29). Another study demonstrated that the selective elimination of neurons located in some cell groups of the BFCN (e.g., Ch1 and Ch2)—via IT-mediated cell targeting—is linked to some types of impaired recognition memory (e.g., spatial recognition memory) (32). Additionally, evidence indicates that cholinergic signaling in the macaque dorsolateral prefrontal cortex is important for spatial working memory, as a cholinergic depletion in this region leads to spatial memory impairment (35). Together, these findings indicate that various cognitive functions are supported by the structural integrity of the cholinergic system. Beyond the structural integrity, evidence suggests that ACh levels are linked with distinct cognitive functions. For instance, it has been shown that transient rises in prefrontal ACh are associated with increased visual cue detection, indicating a link between ACh levels and attention (25). In support, ACh increases, measured via microdialysis, were related to increased task performance (31). Similarly, another study corroborated this link by showing that increased ACh release in cortical and hippocampal areas is associated with cognitive performance on a learning and spatial memory task (33). Furthermore, increasing the level of ACh by donepezil, an acetylcholinesterase inhibitor, has shown to improve attention and vigilance in non-human primates, with apparent dose-dependent pro-cognitive effects (38). Specifically, the dose level for maximally improved attention differed from the dose range that enhanced cognitive flexibility in rhesus monkeys. Enhancement of ACh level by donepezil has shown to improve working memory, comparable to attention enhancements, in non-human primates (39). However, beyond overall increases in ACh, selective manipulations of cholinergic receptors may be relevant for specific cognitive functions. For instance, the stimulation of α4β2-nAChRs in the medial PFC has been linked to enhanced performance in a visual attention task (30). Notably, this effect was stronger when elicited by specifically stimulating the α4β2-nAChRs than via non-selective stimulation by nicotine. Furthermore, a study by Callahan et al. (40) aimed to increase memory performance in young and aged rodents and aged non-human primates by targeting nAChRs selectively, by combining the acetylcholinesterase inhibitor donepezil with a positive allosteric modulator (PAM) of α7-nAChR. While donepezil administered alone increased memory performance significantly in all conditions, it did so only in a dose-dependent manner with the occurrence of dose-limiting side effects, and the α7-nAChR PAM administered alone had no significant effect on memory performance. In turn, the combination of both drugs increased memory performance significantly. In fact, the α7-nAChR PAM increased the effective dose range of donepezil, an effect apparently mediated by α7-nAChR. However, memory performance is not necessarily mediated by nAChRs. A novel, highly selective M4 muscarinic PAM, namely Compound 24, was reported to have beneficial effects on executive functions and memory as well (41). Intriguingly, Thiele and Bellgrove (42) have argued that muscarinic and nicotinic receptors contribute to the neuronal signatures of attention in a cell-type-dependent manner. For example, in the macaque frontal eye field only muscarinic receptors are involved in attentional modulation of broad spiking (putative pyramidal) cells, whereas both muscarinic and nicotinic receptors are involved in attentional modulation of narrow spiking (putative inhibitory) cells.

Correspondingly, human studies applying pharmacological manipulations to the cholinergic system in healthy participants, have highlighted the system's role in facilitating (unimpaired) cognition. For example, the administration of scopolamine, an mAChR antagonist, was associated with a decrease in memory processes, namely impaired paired-associate learning and increased proactive interference (43, 44). Likewise, the administration of mecamylamine, an nAChR antagonist, was linked to impaired short-term working memory processes (45). In contrast, the administration of HTL0018318, an M1-mAChR agonist, facilitated short-term memory and learning in healthy participants (46). Similarly, the administration of nicotine, serving as a nAChR agonist, led to enhanced attention and overall performance in healthy participants (47–49). However, the effect of nicotine administration must be interpreted carefully, since several inconsistent effects have been reported. For example, Newhouse et al. (50) have provided evidence that nicotine enhances attentional, memory, and psychomotor performance in participants with mild cognitive impairment, however, no effect was found in clinician-rated global improvement. In another study, nico. Tine was found to have no effect on several cognitive functions in young healthy participants and even decreased performance on working memory and visual memory in elderly participants (51). Intriguingly, the authors suggest a baseline performance-dependent effect indicated by positive effects of nicotine on cognition in low baseline performers compared to high baseline performers. In line with this study, an “inverted U” dose—response relationship is suggested to reflect the effects of nicotine on cognition. That is, low, sub-threshold doses of nicotine are ineffective in enhancing cognitive functions, whereas higher doses may elicit positive effects, but even higher doses result in no or even detrimental effects on cognitive functions.

We note, however, that various methodological issues may underlie such distinct effects. For instance, it has been put forth that studies failed to consider the influence of profound differences between individuals (i.e., genetic factors) regarding their sensitivity to nicotine or a shift in activation or desensitization of response over time (52). Another notable issue is the failure of some studies to assess baseline-related effects, raising the issue of regression to the mean.

Further evidence for nicotinic modulation of neurophysiological processes in humans has been reported by pharmaco-fMRI studies. For instance, several pharmaco-fMRI studies have demonstrated an increase in task-related activity after nicotine administration in non-smokers and deprived smokers, but not in active smokers [reviewed in (53)]. In line with these findings, nicotine was reported to decrease the activity in regions related to the default mode network, typically associated with task disengagement (53). Beyond increases in task-related activity, administration of nicotine to healthy volunteers has been shown to neuronally modulate subsystems of selective attention, namely the reorienting of visuospatial attention and “alerting” (reflecting the general readiness and increased responsiveness to a target) within the parietal cortex (54). Interestingly, however, on the behavioral level, alerting was not affected by nicotine, whereas reorienting of attention was enhanced by a faster reaction time, indicating that neurophysiological changes might not always be reflected in behavioral measures.

In summary, the reviewed studies demonstrate that targeting specific subreceptors may be more effective for the enhancement of certain cognitive functions than a general increase in ACh transmission. Importantly, the effects of ACh on cognitive functions have to be understood in a dose-dependent way. Furthermore, a clear link between certain cognitive functions and specific receptor subtypes is difficult to establish. While specific receptor subtypes may be linked to some cognitive functions in a cell-type-dependent manner, overlap is also present, indicating functional interactions between mAChRs and nAChRs.

## Cholinergic Dysfunction in Psychotic Disorders

Several lines of evidence indicate that an altered cholinergic system plays a role in the pathophysiology of psychotic disorders, particularly schizophrenia, and may be relevant in eliciting and/or maintaining distinct symptom dimensions, including positive and negative symptoms, and cognitive impairments. In the following, we present evidence obtained with several different techniques that suggest particularly reduced mAChR and nAChR availability and lower BFCN volumes in patients with schizophrenia. Evidence regarding other psychotic disorders is limited.

### Post-mortem Studies

Post-mortem findings provide direct evidence that M1/M4-mAChRs are reduced in several brain regions in patients with schizophrenia, particularly in prefrontal cortices (55–59). Since no similar decrease of M1/M4-mAChRs occurs in bipolar disorders (60) but a decrease in M2-mAChrs has been observed in mood disorders (61, 62), the effect of an M1/M4-mAChRs reduction in schizophrenia may be receptor subtype specific. Post-mortem studies also provide evidence, albeit less clear, for decreased nAChRs availability, especially α7-nAChRs, in several brain regions including the hippocampus and cingulate cortex in patients with schizophrenia (62, 63). Notably, however, other studies observed no reduction of α4- or β2-nAChRs (64, 65).

### Molecular Imaging Studies

In line with the evidence from the post-mortem findings, SPECT studies report reduced nAChR and mAChR availability in patients with schizophrenia. For instance, Raedler et al. (24) used [(123) I] IQNB SPECT to compare mAChR availability between unmedicated patients with schizophrenia and healthy controls. MAChR availability was significantly lower in patients with schizophrenia compared to healthy controls in the cortex, basal ganglia, and thalamus. Furthermore, the mAChR availability in the striatum and frontal cortex were negatively correlated with positive symptoms (i.e., the lower the mAChR availability the more severe the positive symptoms). However, due to the non-specific nature of the tracer, these researchers were not able to determine which of the multiple mAChR subtypes were reduced (19). This is highly problematic when trying to relate such “mechanistic” findings to function, as mAChR subtypes have distinct excitatory and inhibitory activity. Thus, it remains unclear which aspect of ACh transmission is related to positive symptoms. Another SPECT study by D'Souza et al. (23) investigated nAChR availability via [123I] 5-IA SPECT in smokers with and without schizophrenia. Smokers with schizophrenia showed significantly lower β2-nAChR availability in the frontal cortex, parietal cortex, and thalamus relative to smokers without schizophrenia, with the main reduction found in the frontal cortex. In addition, patients with lower β2-nAChR availability had more pronounced negative symptoms. The lower β2-nAChR availability in smokers with schizophrenia may reflect an abnormal desensitization or turnover of nicotine or a deficit in nicotine-induced upregulation (23).

The suggested relationship between negative symptoms and β2-nAChR availability in schizophrenia may explain the high rates of smoking in schizophrenia, supporting the idea of the “self-medication”-hypotheses with a nicotine-induced reduction of negative symptoms and cognitive impairment (66). Further evidence for altered nAChR availability is provided by a cross-sectional PET study using 18F-ASEM, a radiotracer targeting the α7-nAChR, in non-smoking individuals with recent onset of psychosis; both affective and non-affective (67). In detail, Coughlin et al. (67) reported lower availability of the α7-nAChR primarily in the hippocampus of patients with non-affective psychosis compared to healthy controls. Although this reduced availability was quantified in the hippocampus only, the authors presume possible reductions across the whole brain. Crucially, lower α7-nAChR availability was linked to low performance in two cognitive domains (linguistic processing speed and verbal memory) after controlling for age. More evidence for reductions in volume of distribution in the cingulate and frontal cortex, and hippocampus in patients with schizophrenia comes from a PET study using a similar 18F-ASEM radiotracer for targeting α7-nAChR (68). Table 1 provides an overview of the *in-vivo* imaging studies demonstrating alterations of the cholinergic system in psychotic disorders.

**Table 1:** *In vivo* neuroimaging studies demonstrating cholinergic system alterations in psychotic disorders.

**Neuroimaging method**	**Measured brain domain**	**Participants**	**Neuroimaging and statistical results**	**Related symptoms/cognitive function**	**References**
[(123)I] IQNB SPECT	mAChR availability	Schizophrenia patients (*n* = 12) and healthy controls (*n* = 12)	Reduced mAChR availability in frontal cortex of schizophrenia patient [*t*_*(20)*_= 3.29, *p* = 0.004]	Increased positive symptoms only for the frontal cortex (*r* = −0.64, *p* = 0.03) and the striatum (*r* = −0.63, *p* = 0.03)	(24)
[123I] 5-IA SPECT	ß2-nAChR availability	Smokers with schizophrenia (*n* = 11) and comparison smokers (*n* = 11)	Reduced β2-nAChR availability in parietal cortex [*F*_*(1, 20)*_ = 4.73, *p* = 0.04] and thalamus of smokers with schizophrenia [*F*_(1, 20)_ = 5.50, *p* = 0.003]	Increased negative symptoms only for parietal cortex (*r* = −0.61, *p* < 0.05)	(23)
18F-ASEM PET	α7-nAChR availability	Psychosis patients (*n* = 11) and healthy controls (*n* = 15)	Reduced α7-nAChR availability in hippocampus of psychosis patients (*p* = 0.001)	Decreased speed processing (*r* = 0.73, *p* < 0.05) and verbal memory (*r* = 0.75, *p* < 0.05)	(67)
18F-ASEM PET	α7-nAChR availability	Schizophrenia patients (*n* = 6) and healthy controls (*n* = 21)	Reduced α7-nAChR in cingulate cortex and hippocampus (*p* = 0.02)		(68)
Structural MRI	GM volume BFCN	Schizophrenia patients (*n* = 72) and healthy controls (*n* = 73)	Lower GM volume of the BFCN of schizophrenia patients [*t*_(139)_ = 2.5, *p* = 0.01]	Decreased attentional capacity [*r* = 0.31, *p* = 0.01]	(10)

In summary, *in vivo* SPECT and PET studies have consistently demonstrated a downregulation of acetylcholine receptors in patients with psychotic disorders, whereby nAChR downregulation might be linked to negative symptoms, and mAChR downregulation to positive symptoms. Interestingly, a downregulation of both ACh receptor types may be relevant for several cognitive deficits. However, small sample sizes and low tracer specificity in these studies hamper the development of univocal trust in these findings and their generalization. Furthermore, it is unclear whether the described alterations of the cholinergic system are related to the BFCN themselves or rather reflect additional pathophysiological processes. Answering this question is complicated by limited knowledge about the structural integrity of the BFCN and their relationship to other brain regions in patients with psychotic disorders. Such putative alterations can be investigated *in vivo* with structural and functional fMRI.

### Structural MRI Studies

Evidence for structural alterations of the BFCN is limited as only one study has been published to date. This recent MRI study addressed the issue of BFCN structural integrity using voxel-based morphometry (22). Specifically, gray matter (GM) volume differences were evaluated within a cytoarchitectonically defined mask of the BFCN between patients with schizophrenia and healthy controls. This study provided evidence for reduced BFCN volumes in patients with schizophrenia. Crucially, these findings were replicated in a completely independent sample of patients with schizophrenia and healthy controls. The authors also demonstrated that structural alterations of the BFCN were relevant for patients' cognitive impairment, as smaller BFCN volumes mediated performance on an attention task, performed outside the scanner.

### Early Alterations of the BFCN May Relate to Later Risk for Developing Psychosis

There is limited evidence of a link between early alterations of the BFCN and an increased risk of developing psychosis later in life (69).

### Anticholinergic Burden

The available antipsychotic medication for the treatment of psychotic disorders often has anticholinergic effects, beyond the affinity at D2 dopaminergic receptors (70). It has been consistently shown that such anticholinergic properties, the so-called anticholinergic burden, correlate with cognitive impairment in patients with schizophrenia (71, 72). Such findings provide further evidence for a link between cognitive impairment and alterations of the cholinergic system in patients with psychotic disorders.

### Alzheimer's Disease

Psychotic symptoms, including hallucinations and different types of delusions (i.e., of persecution, infidelity etc.) are relatively common in Alzheimer's disease (1). Intriguingly, evidence suggests that the cholinergic system may also be relevant for psychotic symptoms in Alzheimer's disease. For instance, elevated muscarinic M2 binding was shown to be increased in frontotemporal regions of patients with Alzheimer's disease and psychotic symptoms (73). Furthermore, reduced acetylcholinesterase activity has been reported in Alzheimer's disease patients suffering from hallucinations (74). In addition, there is some evidence that the use of cholinesterase inhibitors is linked to an attenuated risk of antipsychotic initiation but larger, better designed studies are needed (75).

### Open Questions

**Figure 1:**
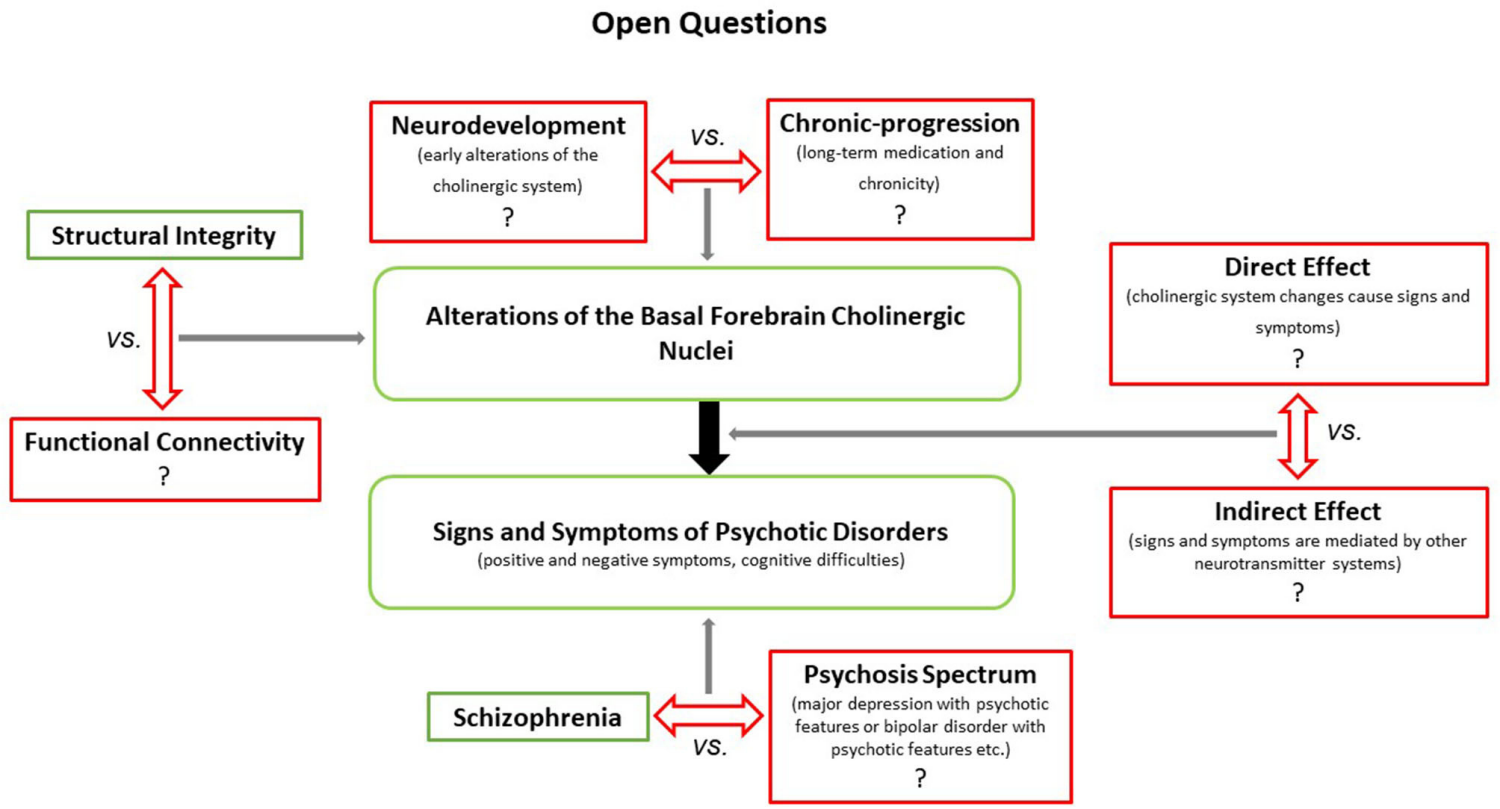
Illustrates open questions concerning the putative role(s) of the BFCN in the generation and maintenance of distinct symptoms in schizophrenia and other psychotic disorders. First, it remains to be determined whether the apparent structural (and potentially functional) changes of the BFCN in patients with schizophrenia reflect effects of neurodevelopmental or chronic-progressive processes. Second, it is unclear whether BFCN alterations induce/maintain symptoms directly (e.g., via attenuated cholinergic transmission) or indirectly (e.g., via interactions with the dopaminergic system). Finally, while recent imaging studies have shown that cholinergic transmission is altered in other psychotic disorders beyond schizophrenia (67), it is unclear whether BFCN alterations are schizophrenia specific or rather ubiquitous across psychotic disorders.

As most of the findings presented above were reported for patients with chronic schizophrenia, it remains unclear whether such effects are a result of neurodevelopmental processes or rather neurodegenerative ones, linked to the trajectory of the disease or treatment effects such as hospitalization or long-term antipsychotic medication. Additionally, it is unclear whether such BFCN alterations are psychosis specific since studies on patients with psychotic disorders other than schizophrenia are scarce. Regarding functional specialization, future studies are needed to determine whether certain subdivisions of the BFCN can be linked to distinct symptom dimensions. This is relevant, as distinct projections and functional specializations of the cell groups contained in the BFCN can be divided into separate functional parts (18). For an overview of the open questions, see Figure 1.

### Future Directions

Resting-state functional MRI is a promising method that may shed some light on the issues presented above but studies are scarce. For instance, although the connectivity of the BFCN has not yet been evaluated in patients with psychotic disorders, recent investigations in healthy participants have demonstrated functional connectivity (i.e., statistical dependencies of BOLD time-series) between the BFCN and several cortical regions, which are organized topographically (76, 77). Briefly, in an ultra-high field study by Yuan et al. (76), the BFCN were parcellated into three clusters that demonstrated rostral to caudal differences in functional connectivity of the BFCN, which were linked functionally with several brain networks. Although this approach demonstrated segregation at specific target regions, overlap was also present. Similarly, Fritz et al. (77) used a two-cluster solution, separating the BFCN into an anterior and posterior part. This solution also revealed findings in line with the functional topography of the BFCN. For instance, the anterior BFCN showed functional connectivity with limbic networks and anterior-medial temporal cortices, as well as midline and posterior-medial temporal subsystems of the default mode network (DMN). Correspondingly, the posterior BFCN cluster also showed functional connectivity with the anterior-medial temporal limbic network but differed from the anterior BFCN in showing selective functional connectivity with additional networks and/or hubs (e.g., the posterior ventral attention network, lateral-temporal parts of the DMN, and the ventrolateral somatomotor network). These studies demonstrate proof of concept and beg the question of whether and how such functional connectivity patterns may differ in patients with psychotic disorders. Furthermore, investigating the relationships between putative BFCN connectivity changes and clinical measures may shed more light on the BFCN's functional specializations and their role concerning distinct symptom dimensions in psychotic disorders.

## Role of Cholinergic Agents as Therapeutic Agents

Based on the SPECT and PET results as well as the postmortem findings described above, mAChRs and nAChRs (i.e., α7/β2-nAChRs, and M1/M4-mAChRs) are emerging as targets for developing novel treatments for psychotic disorders. However, addressing receptor subtypes selectively and without dose-limiting adverse effects appears challenging to date (78).

For instance, administering nicotine to patients with schizophrenia has been shown to increase performance on working memory and selective attention tasks (79). It has also been shown to improve accuracy and reaction time during sustained visual attention tasks (80). It is worth mentioning that nicotine binds with high affinity to α4β2-nAChR and with a much lower affinity to α7-nAChR, the subtype which is rather associated with schizophrenia (68). However, a recent meta-analysis by Recio-Barbero et al. (81) points out that the current evidence of effects of administering α7-nAChR agonists for schizophrenia—as an add-on to antipsychotic treatment in tackling cognitive and negative symptoms—is too weak to consider as an effective treatment approach. Specifically, the authors note no significant effects on cognitive impairment and only small effects on negative symptoms.

Similarly, xanomeline, a M1/M4-mAChR agonist, has been reported to improve cognitive functions in patients with schizophrenia (21). Remarkably, a recent clinical trial has shown that treatment with xanomeline can also attenuate psychotic symptoms in patients with schizophrenia (16). However, both studies were accompanied by several cholinergic adverse events, such as vomiting, diarrhea, and nausea. One reason might be the non-highly-selective effects of xanomeline on mACHRs (78). Although these effects were reduced when xanomeline was combined with trospium-chloride, a peripherally restricted pan-muscarinic antagonist, larger and longer trails are required to determine the efficacy and safety of xanomeline-trospium. Furthermore, it is still unclear if both M1 and M4 mAChR agonism is necessary for xanomeline's efficacy (16). Indeed, Moran et al. illustrate that a better understanding of the pharmacological properties, important for the efficacy of such selective mACHR modulators and responsible for adverse effects, is imperative for facilitating more successful clinical trials. A similar conclusion was reached by Newhouse (82) with respect to nAChR modulation.

Other issues worth mentioning in the development of cholinergic agents are translational challenges. Despite promising preclinical trials investigating the role of cholinergic agents in attenuating cognitive impairment in schizophrenia, the clinical applicability of many studies remains limited. For instance, a meta-analysis by Lewis et al. (83) discusses translational challenges with respect to α7-nAChR agonism. For example, α7-nAChR genetic, pharmacological, and expression differences between rodent and human may mediate the observed differences between preclinical studies and clinical trials. Furthermore, discrepant dosing paradigms between rodent preclinical studies and clinical trials are also problematic. In contrast to acute dosing in large clinical trials, receptor desensitization or functional antagonism may lead to deviating effects by the same dose following chronic administration, indicating that acute dosing may not predict the effects of chronic dosing. Additionally, the inverted U-shape curve of dosing on cognitive tasks and the wide range of receptor activation complicates the specific choice of dose in clinical trials. Finally, it is worth mentioning that acute dosing is not affected by drug intolerance or side effects that develop over time. Such issues become highly relevant in phase II and III clinical trials (82). For instance, EVP-6124 (encenicline), a α7-nAChR agonist had shown promising phase II results in improving the cognitive functions of patients with schizophrenia. However, a large phase III trial failed (84) mainly due to unexpected severe adverse effects.

## Conclusion

In conclusion, strong evidence exists for cholinergic dysfunctions being relevant for psychotic disorders, particularly schizophrenia. Specifically, alterations in ACh transmission demonstrated by reduced availability of specific subtypes of nAChRs and mAChRs may contribute to signs and symptoms of psychotic disorders, such as cognitive impairments. Although it is unlikely that certain symptom dimensions of schizophrenia result from dysfunctions of the cholinergic system alone, recent evidence strongly suggests that the cholinergic system is highly relevant for the pathophysiology of psychotic disorders. However, it remains unknown whether alterations in ACh neurotransmission are related to structural abnormalities of the BFCN or altered connections between the BFCN and other regions of the brain. Similarly, it is unclear whether the structural alterations are ubiquitous in psychotic disorders or rather schizophrenia specific, although there are indications for the former. Additionally, it remains to be determined when these alterations arise and whether they reflect neurodevelopmental or chronic-progressive processes. To address these gaps, future studies are needed to investigate the BFCN in different psychotic disorders and distinct stages of the disorder—for example, between patients with first episode psychosis compared to those with a chronic trajectory. Furthermore, additional imaging methods such as resting-state fMRI should be employed to study BFCN connectivity in psychosis. The findings of such future studies will be paramount in guiding the development of new treatment options for psychotic disorders and potentially also for patients' cognitive impairments.

## Author Contributions

MA and SB conceived the idea, outlined the review, and approved the submitted version of the manuscript. SE and MA wrote the manuscript. LF, AK, HR, V-NT, and SB gave constructive feedback and made editorial contributions. All authors contributed to the article and approved the submitted version.

## Funding

This work was funded by ERA-NET NEURON Project Linking synaptic dysfunction to disease mechanisms in schizophrenia - a multi-level investigation (SYNSCHIZ) (No. 179602).

## Conflict of Interest

The authors declare that the research was conducted in the absence of any commercial or financial relationships that could be construed as a potential conflict of interest.

## Publisher's Note

All claims expressed in this article are solely those of the authors and do not necessarily represent those of their affiliated organizations, or those of the publisher, the editors and the reviewers. Any product that may be evaluated in this article, or claim that may be made by its manufacturer, is not guaranteed or endorsed by the publisher.
